# Magnetic order and competition with superconductivity in (Ho-Er)Ni_2_B_2_C

**DOI:** 10.1088/2053-1591/abc998

**Published:** 2020

**Authors:** Suleyman Gundogdu, J Patrick Clancy, Guangyong Xu, Yang Zhao, Paul A Dube, Tufan C Karalar, Beong Ki Cho, Jeffrey W Lynn, M Ramazanoglu

**Affiliations:** 1 Physics Engineering Department, Istanbul Technical University, 34469, Maslak, Istanbul, Turkey; 2 Department of Physics and Astronomy, McMaster University, Hamilton, Ontario L8S 4M1 Canada; 3 NIST Center for Neutron Research, National Institute of Standards and Technology, Gaithersburg, Maryland 20899, United States of America; 4 Brockhouse Institute for Materials Research, Hamilton, ON L8S 4M1, Canada; 5 Electronics and Communication Engineering Department, Istanbul Technical University, 34469, Maslak, Istanbul, Turkey; 6 Gwangju Institute of Science and Technology, GIST, Republic of Korea

**Keywords:** superconductivity, magnetic materials, neutron scattering, magnetization measurements

## Abstract

The rare earth magnetic order in pure and doped ${Ho}_{\left(1-x\right)}{Er}_{x}{Ni}_{2}B_{2}C$ (x = 0, 0.25, 0.50, 0.75, 1) single crystal samples was investigated using magnetization and neutron diffraction measurements. Superconducting quaternary borocarbides, $R{Ni}_{2}B_{2}C$ where *R* = Ho, Er , are magnetic intermetallic superconductors with the transition temperatures~10 K in which long range magnetic order develops in the same temperature range and competes with superconductivity. Depending on the rare earth composition the coupling between superconductivity and magnetism creates several phases, ranging from a near reentrant superconductor with a mixture of commensurate and incommensurate antiferromagnetism to an incommensurate antiferromagnetic spin modulation with a weak ferromagnetic component. All of these phases coexist with superconductivity. RKKY magnetic interactions are used to describe the magnetic orders in the pure compounds. However, the doping of Er on Ho sites which have two strong magnetic moments with two different easy directions creates new and complicated magnetic modulations with possible local disorder effects. One fascinating effect is the development of an induced magnetic state resembling the pure and doped *R*_2_CuO_4_ cuprate with R = Nd and Pr.

## 1. Introduction

Rare earth (*R*) nickel borocarbides $R{Ni}_{2}B_{2}C$ have managed to stay a current research topic for more than 25 years. The reason for this longtime attraction is related to their magnetic low temperature states which occur and compete on an equivalent energy scale with superconductivity (SC) [1, 2]. Magnetic order in superconductors has been a topic of keen interest for over a half century [3], but quaternary borocarbides are of special interest for two main reasons. First, the magnetic order and SC occur in the same temperature range. Secondly, high quality single crystals with a wide range of rare earth ions *R* are readily available for investigation. In particular, the choice of *R* element allows a wide range of magnetism to emerge, both commensurate and incommensurate antiferromagnetic (AFM) modulations to a ferromagnetic (FM) state [4–7]. These magnetic states naturally compete with SC and create exotic phenomena with long range order coexisting and competing with the superconducting state. This competition is important when SC, which is a q=0 ordered magneto-electronic state, interplays with magnetism which usually orders along a q≠0 vector (except for FM) [8]. The availability of high quality single-crystal samples for all *R* members of the $R{Ni}_{2}B_{2}C$ family makes this class of materials prototypes for SC-magnetic interplay investigations. The magnetic interactions in $R{Ni}_{2}B_{2}C$ emerge via the Ruderman-Kittel-Kasuya-Yosida (RKKY) model interactions that couple the local spins to the itinerant conduction electrons, where the Fermi surface can play a role not only in the magnetic order that emerges but also in the phonon dynamics associated with the superconducting pairing [9, 10].

The $R{Ni}_{2}B_{2}C$ crystal structure is body centered tetragonal (I4/mmm at room temperature) for the R=Er and Ho system of interest here. The unit cell dimensions are very close to each other (a~3.518Å,c~10.527Å for *R* = Ho and a~3.502Å,c~10.558Å for R=Er) readily allowing substitutions across the entire concentration range [5]. In the pure *R* = Ho case, the Ho moments are coupled ferromagnetically in the a−b plane, forming FM sheets which are antiferromagnetically stacked along the *c*-axis. This pattern of moments produces antiferromagnetic modulations with $q_{c}=0.915c^{*}$ that exists over a short temperature interval upon initial ordering and then a commensurate antiferromagnetic (C-AFM) structure which coexists with SC. The associated ordering temperatures for the development of the C-AFM and the SC phases coincide at the same values of T ~ 8 K[11, 12]. In addition, the first single crystal studies revealed an additional incommensurate (IC) *a*-axis modulation over a narrow temperature range between 5K⩽T⩽7K with a wave-vector of $q_{a}=0.585\;a^{*}$ associated with the Fermi surface [11]. Many additional details of the magnetic structures have been elucidated via high resolution neutron and x-ray investigations on single crystals, including a small structural distortion from tetragonal to orthorhombic settings [13–15]. ${ErNi}_{2}B_{2}C$, on the other hand, develops a transversely polarized spin density wave modulation which is always incommensurate, with a wave-vector of $q_{a}=0.55\;a^{*}$ at $T_{N}\sim 6K$ [6]. This means the easy direction for the *Er* moments is along the *b*-axis. As the order developed with decreasing temperature, higher order reflections were observed reflecting the squaring up of the spin density wave order. More interestingly, a net magnetization was found to develop below 2.3 K due to a distortion of the spin density wave, breaking the time reversal symmetry in the system [4, 6, 8, 16]. The pure magnetic phases and the superconductor states for R=Er and Ho in $R{Ni}_{2}B_{2}C$ develop in the vicinity of the same temperature and hence energetics. This makes the doped compounds even more interesting to investigate the coupling between the SC and magnetism. The substitution of Ho sites with Er ions (or vice versa) possibly influences the RKKY exchange interactions and changes the type of order with respect to the pure material. This would also change the crystal electric fields (CEF) and their coupling with the RKKY modulations. These newly obtained states for ${Ho}_{\left(1-x\right)}{Er}_{x}{Ni}_{2}{\;B}_{2}C$ may show new IC and/or CAFM modulations or even destroy some of the original ordering seen in pure systems.

In this study we focus on magnetism, superconductivity and the interplay of these states within ${Ho}_{\left(1-x\right)}{Er}_{x}{Ni}_{2}B_{2}C$ for x = 0, 0.25, 0.50 and 0.75 single crystals using magnetic susceptibility and triple-axis neutron diffraction techniques.

## 2. Materials and methods

Single crystals of $R{Ni}_{2}B_{2}C;R={Ho}_{\left(1-x\right)}{Er}_{x}$ with x = 0, 0.25, 0.50 and 0.75 were grown using a slow-cooling self-flux technique [17]. These samples contain isotopically enriched ^11^B isotopes to minimize the neutron absorption of natural boron. The magnetization measurements were performed at the Brockhouse Institute for Materials Research (BIMR) at McMaster University using a Quantum Design Magnetic Properties Measurement System (MPMS). Single crystal samples having flat surfaces for crystallographic a−b planes were aligned so that an *a*-axis is parallel to the applied field direction. The neutron diffraction experiments were conducted at NIST Center For Neutron Research (NCNR). Thermal triple-axis BT-9 and double focusing tripleaxis BT-7 instruments [18] and NG-5 SPINS cold triple-axis neutron spectrometers were used. Both closed cycle and cryogen cryostats were used for performing temperature scans on different instruments. Most of the measurements were performed to the lowest possible temperature (in the vicinity of 1.5 K). The incident neutron energy was set to 14.7 meV for BT-7 and BT-9 and 5 meV for NG-5 measurements. The beam-line BT-9 was used with a 40-40-PG-Sample-PG-40-open collimation setting. Here, the PG term stands for the pyrolytic graphite filters used before and after the sample during the experiment. Measurements on the NG-5 instrument were performed using a cold Beryllium Filter (BE) with Guide-BE-80-Sample-80-BE-single detector collimation. For the BT-7 measurements a position sensitive detector (PSD) with 80-PG-80R-Sample-PSD collimation was employed. The map of magnetically interesting reciprocal space was collected using the PSD. The PSD covered 6 deg 2θ range and by setting it successively at 12°, 17° and 22°, counts over a 2θ range from 9° to 25° could be collected. For each PSD setting, the sample θ was rotated from 0° to 100° in 0.25° steps giving 401 sample angles. This gave the coverage of reciprocal space shown below. Scans in reciprocal space are presented in the reciprocal lattice units of $H=\frac{2\pi }{a}$ and $L=\frac{2\pi }{c}$ where a is ~3.5Å and c is ~10.5Å. Our aim was to study the magnetic structures and the superconductivity of each sample depending on the x amount.

## 3. Results

**Figure 1.**
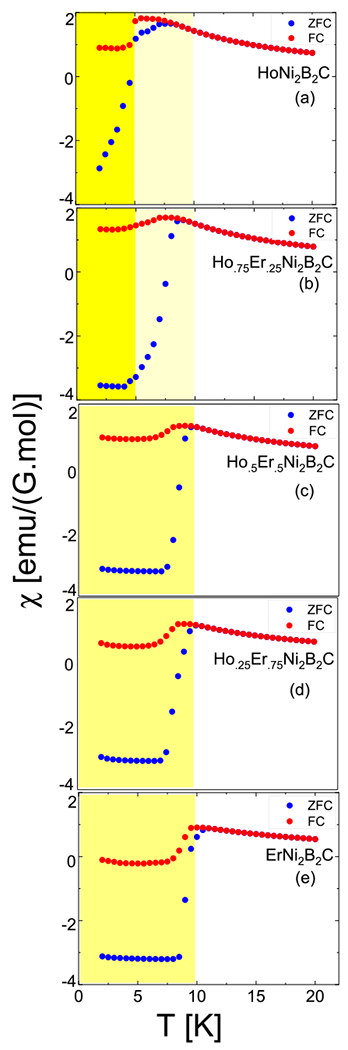
Susceptibility measurements for all samples. The yellow regions mark the superconductivity where diamagnetism is partially or completely observed. Very light yellow regions especially for panel (a) and partially in panel (b) indicate the nearly reentrant superconductivity region in pure ${HoNi}_{2}B_{2}C$ and $Hol-x{Er}_{x}{Ni}_{2}B_{2}C$ where x = 0.25. The region with perfect diamagnetism is shown with one tone darker yellow color. The offset temperature of Er doped samples marked with dark yellow regions increases from 4.5 K to 9.5 Kas x increases from 0.25 to 1 in samples as shown in panels (b) to (e). Field cooled measurements were conducted under an applied magnetic field of 1 mT which was held parallel to the *a*-axis of the samples. (Note, 1 emu = 1 × 10^−3^Am^2^).

In order to see the superconducting phase and to determine the transition temperatures as functions of x, we performed a series of DC magnetization measurements, shown in figure 1. The field cooled (FC) and zero field cooled (ZFC) measurements were conducted for ~2⩽T⩽20K range. An applied field of 1 mT was used for FC measurements, and was also used after zero field cooling. Each crystal shows regular cleavage sharp corners pointing along one of the principal axis directions on the clean a−b crystallographic plane surfaces. This was confirmed by Laue pictures which are not shown here. Thus for the magnetic measurements, the field was applied along [100] (or [010]) within ±5° accuracy.

The first common property of all data shown in figure 1. is the paramagnetic behavior above T>10K and the net difference in signal between two different cools. Below 10 K, the diamagnetic signal of the SC phase becomes dominant. The signal difference between ZFC and FC can be explained by the remnant field trapped in the sample in FC cycles which screens the diamagnetic signal. The FC saturated low temperature susceptibility remains positive for all samples except for pure ${ErNi}_{2}B_{2}C$. This is seen in panel (e). There is a net decrease in this value as the Er content increases. However, the positive susceptibility (up to ${Ho}_{0.25}{Er}_{0.75}{Ni}_{2}B_{2}C$ in panel (d)) is evident for 1 mT being enough to penetrate the SC region by partially destroying the diamagnetism and creating possible flux pinning in the samples. In other words, the observed lower critical magnetic field values change as a function of Er concentration and possibly flux pinning effects are being observed. This is in agreement with the value obtained for pure ${HoNi}_{2}B_{2}C$ [19]. In panel (a) the reentrant behavior of the superconducting phase can be seen with a soft decrease in susceptibility at ~8 K. This is followed by a sharp decrease at ~5 K where the diamagnetic signal is obtained indicating the end of the reentrant region and the start of the SC phase. The FC magnetization signal has a sudden decrease in the intensity at the same temperatures. This is valid especially for the pure ${HoNi}_{2}B_{2}C$ data shown in panel (a) of figure 1. The reentrant region disappears as the Er content increases in the other samples. This is shown with the 25% Er sample shown in panel (b). ZFC data shows a saturated diamagnetism for all Er containing samples. Also, the slow decrease in susceptibility signal seen in pure ${HoNi}_{2}B_{2}C$ becomes sharper as the Er content increases. Thus while the onset for SC seen in panel (b) is T~8K for the 25% Er sample, it becomes ~10 K for pure ${ErNi}_{2}B_{2}C$ shown in panel(e). There is a net increase in the temperature values from T~4.5K to T~9.5K for a fully ordered diamagnetic region from 25% Er doped to pure ${ErNi}_{2}B_{2}C$ sample. For the ${Ho}_{0.75}{Er}_{0.25}{Ni}_{2}B_{2}C$ data shown in panel (b), one can suggest the existence of a weak reentrant behaviour for a very small temperature range, however, this entirely disappears for ${Ho}_{0.5}{Er}_{0.5}{Ni}_{2}B_{2}C$.

**Figure 2.**
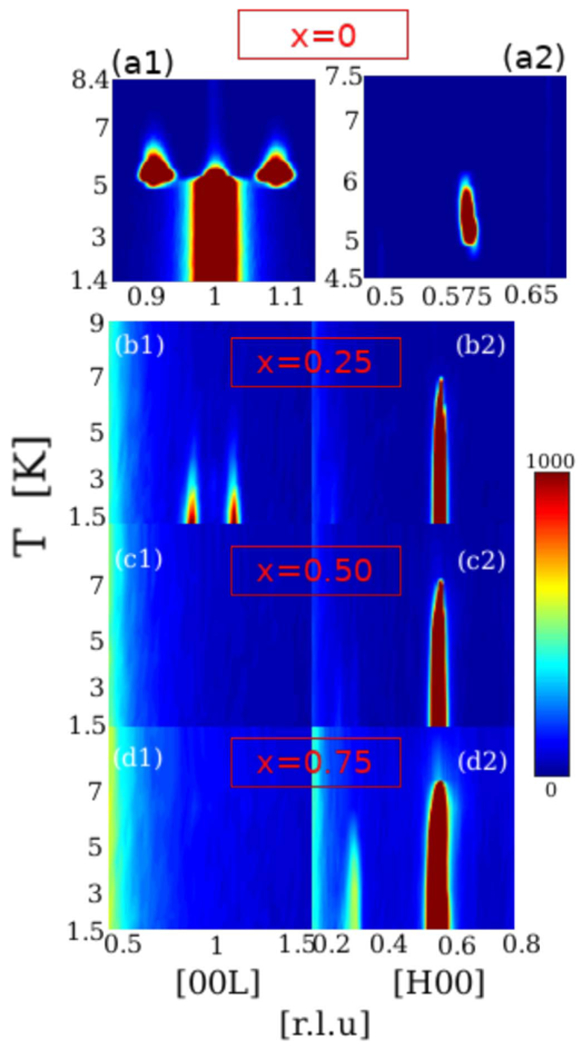
Systematic temperature study of magnetic reflections along [00L] and [H00] directions. The pure system $x=0HoNiB_{2}C$ has one commensurateAFMmodulation at (001) and two incommensurate satelliteAFMmodulations with (001 ± *δ*) where δ=0.09 shown in panel (a1). It also has one incommensurate peak at (0.58 0 0) shown in panel (a2). The same study with a wider region is given for Er containing samples for x = 0.25 (panel b(1) and b(2)), x = 0.50 (panel c(1) and c(2)) and x = 0.75 (panel d(1) and d(2)), in order. Here, each neutron diffraction data set shown for panel (b1, b2) to panel (d1, d2) for Er doped samples is focused on the region of interest shown in panel (a1) and (a2) with wider scattering range. Therefore, the development and disappearing of IC andC magnetic reflections in doped samples can be compared with the pure ${HoNi}_{2}B_{2}C$ behavior.

**Figure 3.**
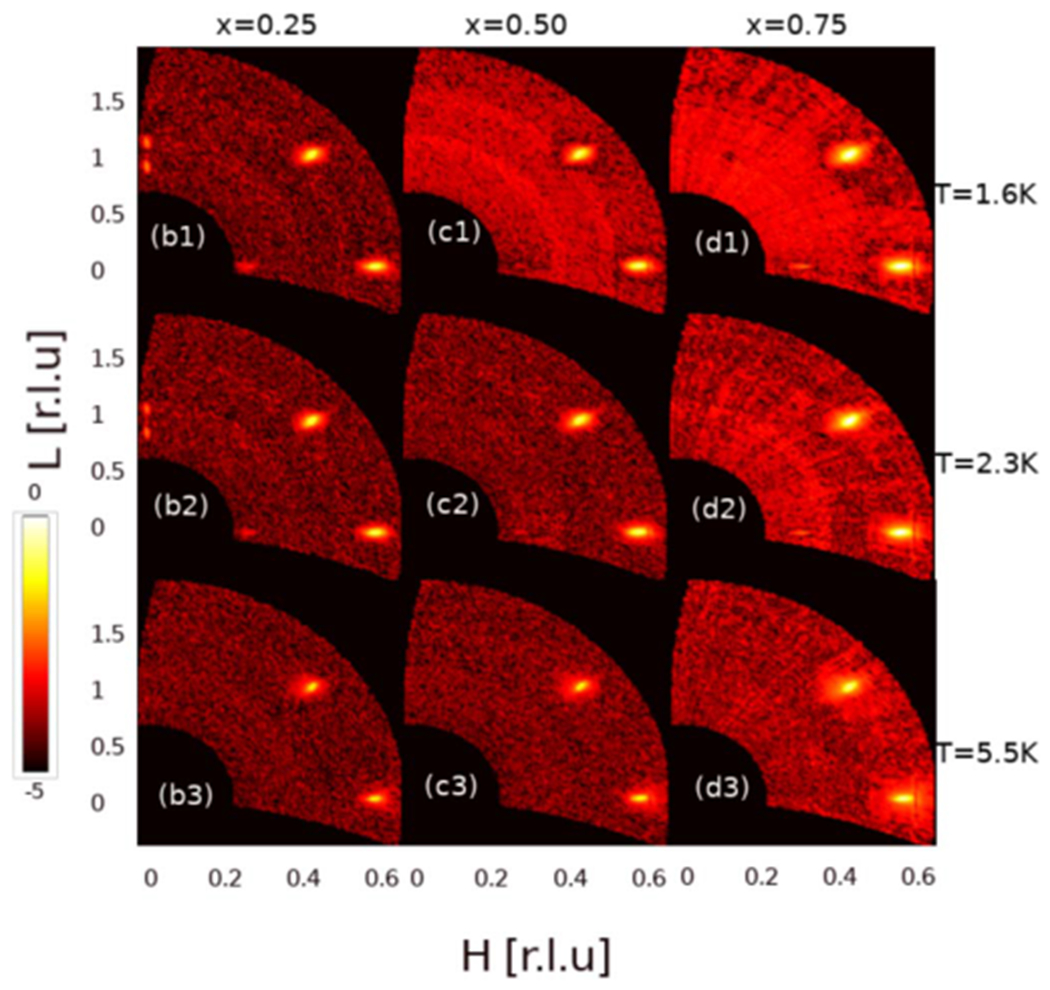
Magnetic neutron diffraction measurements of scattering plane (H0L). These data were obtained using a position sensitive detector (PSD) on the BT-7 spectrometer. Three different temperatures shown on the right side of the panels are used in this study. Each panel is built up by concatenating 3 different scans to form foreground, as explained in the text. T = 9Kdata as background for each sample (not shown) is subtracted from each foreground data and the negative intensity values due to statistical deviations were discarded. A logarithmic intensity scale is used, enabling weak reflections to be seen together with strong reflections. For the ease of reading and comparing, the same order for labeling used in figure 2 is continued in this figure.

**Figure 4.**
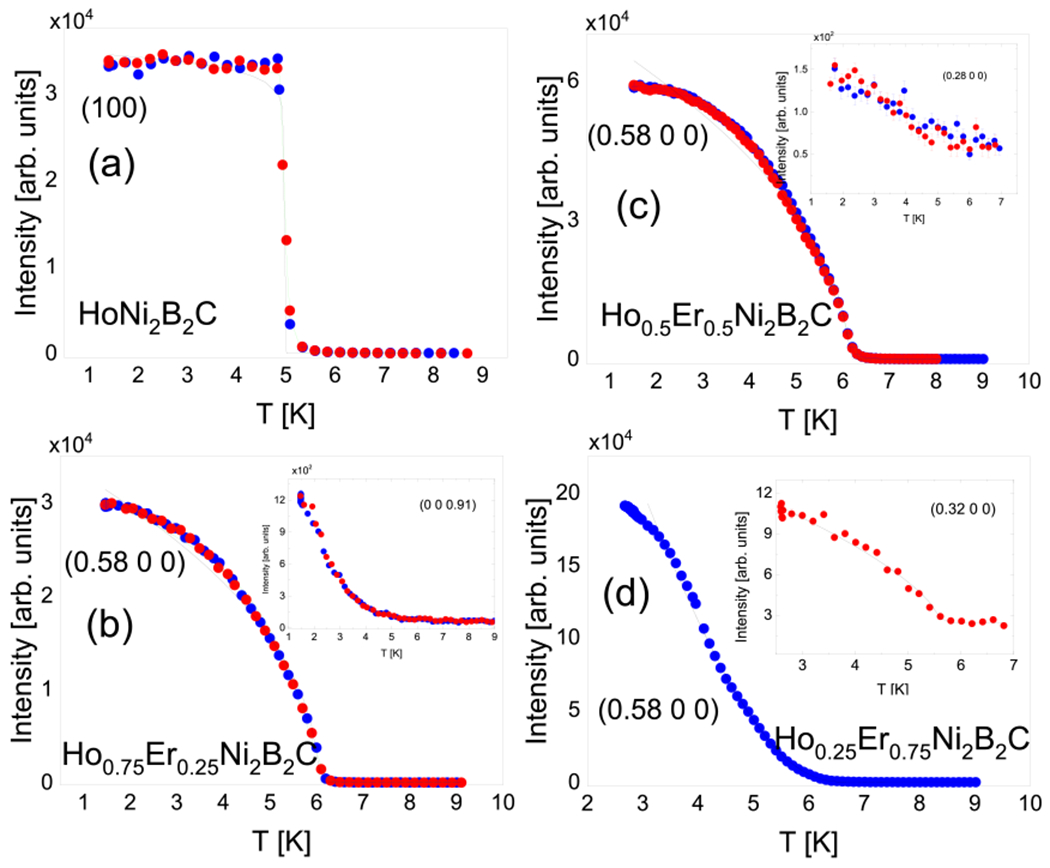
Order parameter fit analyses for different magnetic peaks for x = 0, 0.25, 0.5 and 0.75 samples. The fit results given in table 1 are shown with black and green colored lines. The red and blue colored data shows the heating and cooling, respectively. Error bars represent one standard deviation.

Neutron diffraction measurements along [H00] and [00L] directions centered at H = 0.5 and L = 1, respectively, are shown in figure 2. The main goal for these measurements was to study the Er doping effect as a function of temperature taking the pure ${HoNi}_{2}B_{2}C$ as the reference magnetic system. The temperature dependence of the magnetic reflections shown in panel (a1) and (a2) is for the pure ${HoNi}_{2}B_{2}C$ sample. There are 3 IC peaks which are the characteristic of AFM modulations valid for pure ${HoNi}_{2}B_{2}C$ shown in figure 2 panel (a1) and (a2). Two of these peaks occur as satellite peaks to the long-range C-AFM order shown in panel (a1). The C-AFM peak on (001) behaves as an order parameter (squared) for pure ${HoNi}_{2}B_{2}C$ [12]. The satellite peaks in this direction start to order at almost the same temperature as with the IC modulation seen on (0.5800) in panel (a2). These 3 IC-AFM reflections are observed between 6 K to 5 K. At T ~5K the C-AFM peak which is shown in panel (a1) on (001) starts to order while at this temperature the IC-modulations start to dissolve altogether. This temperature coincides with the diamagnetism oberserved at ~5 K for pure ${HoNi}_{2}B_{2}C$ discussed previously as shown in figure 1. The commensurate magnetic modulation intensity shows a discontinuous order. The temperature maps shown in these two panels serve as the reference for magnetic behaviour of other samples which contain some amount of Er. When the Er content is just x = 0.25 in the ${Ho}_{\left(1-x\right)}{Er}_{x}{Ni}_{2}B_{2}C$ the C-AFM phase is totally destroyed and IC magnetic modulations along L direction order at ~4 K, shown in panel (b1). This temperature is lower than the ordering temperature of pure ${HoNi}_{2}B_{2}C$. Surprisingly, the wave-vectors for these IC modulations are essentially the same as the parent compound, while the intensities keep increasing with decreasing to the base temperature. The IC modulation seen on (0.58 0 0) for the pure ${HoNi}_{2}B_{2}C$ sample still exists for x = 0.25 and other doped levels. For x = 0.25 and the other doped samples, this magnetic reflection behaves as a prominent order peak describing an IC-AFM order shown in panels of figures 2–4.

In panel (c1) and (c2) we see the doping effect of more Er which changes the magnetic behaviour even further away from the reference. The most crowded and thus most interesting scattering plane shown in panel (a1) for the pure system is now empty, without any reflection for x = 0.5. This means that all magnetic moments in the x = 0.5 sample aligned themselves with a modulation along the H-direction, resembling the pure ${ErNi}_{2}B_{2}C$ system. So the spin-density wave (SDW) modulation originally belonging to pure ${ErNi}_{2}B_{2}C$ becomes more pronounced than the AFM orders seen in pure ${HoNi}_{2}B_{2}C$. This behaviour continues for the x = 0.75 sample and this is shown in panel (d1) and (d2). Different from all other samples, for the x = 0.75 sample there is another IC magnetic development at the (0.32 0 0) position. This peak’s intensity increases at low temperatures shown in panel (d2) and the details are given below. Thinking that the origin of this new type of reflection might be a higher order satellite, specifically 3rd harmonic modulation peak seen in pure ${ErNi}_{2}B_{2}C$, we performed position sensitive diffraction (PSD) measurements in the [H0L] magnetic scattering plane to find all possible magnetic Bragg peaks in this plane.

The magnetic modulations studied in temperature scans in the previous figure, are now shown in figure 3 as diffraction from the [H0L] scattering plane for 3 different temperatures. These measurements were conducted using a PSD on the BT-7 instrument for 3 different Er containing samples, x = 0.25, 0.50 and 0.75. For each study, the magnetically important region which is mostly the first Brillouin zone and some part of second one, forming a foreground, was investigated at T = 1.6 K, 2.3 K and 5.5 K. The T = 9 K data were used for each sample as background (not shown) and subtracted from the data. A logarithmic intensity scale is used to reveal the weak intensity peaks. Therefore the data shown in each panel in figure 3 contain solely magnetic intensities. These 3 temperatures were chosen in order to show a more inclusive view of scattering both in the ordered phases and during transitions. 1.6 and 2.3 K data are used to determine the effect of weak FM order seen in pure ${ErNi}_{2}B_{2}C(x=1)$ samples while the 5.5 K data are important to isolate the IC ordering especially valid for pure samples. The satellite peaks at (0 0 0.91) and (0 0 1.09) indicating IC-AFM modulations were seen for the x = 0.25 sample (panel (b1)) at T = 1.6 K while they don’t exist for other Er dopings at this temperature. As temperature increases towards T = 5.5 K these peaks lose their intensities and disappear. This is shown in panel (b2) at T = 2.3 K and in panel (b3) at T = 5.5 K. Interestingly, there is an IC peak appearing at (0.28 0 0) best observed for x = 0.25 shown in panels (b1) and (b2). This peak appears for the x = 0.50 sample at T = 1.6 K and 2.3 K but the intensity is weak compared to the ones for x = 0.25. The dominant peaks for all samples occur at (0.58 0 0) and (0.42 0 1) arising from the scattering in the 1st magnetic Brillouin zone. These two peaks exist at all temperatures, shown in figure 3.

The magnetic intensities shown in figures 2 and 3 have been studied as a function of temperature with finer data sampling in both cooling and heating directions. For this study the magnetic reflections having order parameter like profiles were selected. The results are shown in figure 4. In panel (a) of this figure, the (001) magnetic reflection which is the C-AFM modulation wave-vector for the pure ${HoNi}_{2}B_{2}C$ is shown. The phase transition occurs at T~5K with a 1 st order phase transition character. This is evident from the sudden increase in the observed intensity. The black line in this figure represents the (square of the) order parameter power law fits which are as

(1) $$I(T)\sim {\left(1-\frac{T}{T_{N}}\right)}^{2\beta }$$

(2) $$I(T)\sim \int G\left(T_{N},\Gamma \right){\left(1-\frac{T}{T_{N}}\right)}^{2\beta }dT$$

where $T_{N}$ is the Néel temperature and the fitting parameter of these equations. A more complicated power law fit including a Gaussian to present a distribution of transition temperatures centered on the transition temperature $T=T_{N}$ was also used in the analyses. This is given in equation (2) and the result of its fit is shown in figure 4. The calculated full-width-at-half-maximum (FWHM) obtained from the Gaussian line can be used as another estimate for the strength of the broadening of the phase transition as well as with β. Equation (2) is needed to capture the smearing effects during transition which are possibly created by the existing disorder sources [20, 21].

## 4. Discussion and conclusion

In our samples two rare-earth magnetic elements of Er and Hohave different CEF which act on one another to create some sort of disorder effects. Table 1 shows the results of the fits for these analyses. As seen from the panels of figure 4 and the corresponding values given in table 1, the order parameter exponent β increases as the Er concentration increases. The IC peaks lying on the (0.58 0 0)magnetic reflection exhibit an order parameter for the two samples of x = 0.25 and 0.50. However, the peak profile for x = 0.75 differs slightly from this, shown in panel (d). The fitted β value becomes larger than the mean field value 0.5 for this reflection. At this point another magnetic reflection also catches some attention. The IC modulation which is the satellite peak of pure ${HoNi}_{2}B_{2}C$ at (0 0 0.91) does not disappear below T = 5 K like it does in the pure system. This was shown in figure 2 and the temperature behaviour of this peak is shown in detail in the inset of panel (b) in figure 4. This I(T) profile can be interpreted as induced magnetism [22]. The same argument is also valid for the peak profile of the x = 0.75 sample. In addition, the peak profile shown in the inset of panel (c) suggests a growing order like behavior for the (0.28 0 0) IC satellite. Finally, the (0.32 0 0) peak profile shown in the inset of panel (d) can be fit to a power law and the results are given in table 1.

The critical exponent analysis results are shown in table 1. According to the results the pure ${HoNi}_{2}B_{2}Clong$ rangeAFMorder obtained from the magnetic [100] reflection is a 1st order phase transition. This has been previously obtained and can also be visibly confirmed with our results, figure 4 panel (a) [2, 14]. With our analysis, the power law exponent β is fit to nominally zero which is the hallmark of a discontinuity. As the Er amount increases the RKKY exchange interactions between Er and Hoions including a possible local frustration on each other’s magnetic modulations drive the order character towards the 3D-XY exponent values (β=~0.34) seen for the x = 0.50 results. This argument is fortified by values obtained for the x = 0.25 sample where the obtained β value is in the vicinity of those documented for 2D-XY (β=~0.13) frustrated systems [23, 24]. When x = 0.75 is considered, the magnetic order observed on the (0.58 0 0) magnetic reflection is no longer a conventional order parameter profile. Instead it suggests an induced magnetic order with possible fluctuations where the intensity profile resembles the induced *R* moments under the influence of Cu order seen in $R_{2}{CuO}_{4},R=Nd$ and Pr for example [22]. The induced magnetic order argument is also supported by the two other magnetic reflections shown in the insets of panel (b) and (c) in figure 4 for x = 0.25 and 0.50, respectively. Weremark that this clear induced order implies strong coupling between the two order parameters, which in the case of cuprates mentioned above occurs because the orderings have the same symmetry. This contrasts sharply with ${Sm}_{2}{CuO}_{4}$, for example, where the Cu and Smorder parameters are different and no significant coupling is observed [25]. There are also IC magnetic reflections. In addition, the frustration between the Hoand Er moments can yield new IC magnetic orders such as the one shown with a power law fit in the inset in panel (d) of figure 4. We see that doping Er into ${HoNi}_{2}B_{2}C$ with 25% which is the smallest amount in this study, entirely suppresses the commensurateAFMlong range order in the pure parent compound. Doping with Er also changes the IC-AFM temperature dependence. When the magnetization and susceptibility results are compared, we can conclude that superconductivity and the IC magnetic orders do not strongly compete with each other. Instead they tend to order at low temperatures. With x = 0.50 and higher values the overall magnetic intensity profiles start to look like the ones observed for pure ${ErNi}_{2}B_{2}C$ with no magnetic reflection along the L-direction and with only IC intensities in the H-direction. For x = 0.75 a power law behavior becomes insufficient to capture the overall intensity temperature profile shown in panel (d) of figure 4. The peak profile shown for those IC magnetic reflections have continuous increase in the intensity of the magnetic peak at the low temperature region faster than usual order parameter behavior. This is seen especially for (0 0 0.91) and (0.28 0 0) reflections for x = 0.25 and x = 0.50 samples, respectively. These IC magnetic peak profiles clearly suggest the existence of induced magnetism. The low temperature intensity of (0.58 0 0) peak for the x = 0.75 sample also confirms this behavior as well. Er (or Ho) magnetically ordered sublattices induce each other with slightly different temperature dynamics, and the overall magnetic profiles become similar to the ones previously studied in pure and doped $R_{2}{CuO}_{4},R=Nd$ and Pr.

In this study Er doped ${Ho}_{1-x}{Er}_{x}{Ni}_{2}B_{2}C$ single crystal samples with x = 0.25, 0.50 and 0.75 were investigated usingMPMSand triple-axis neutron diffraction techniques. Further studies using small angle neutron scattering to investigate the effect of the magnetic order on the Abrikosov lattice would be an interesting new avenue to pursue.

## Figures and Tables

**Table 1.** Order parameter fit results. The results of two power law fits shown by black and green lines in figure 4 are given. The units of Néel order temperature $T_{N}$, the superconducting critical temperature $T_{C}$ and the FWHM (Γ) are Kelvin.

Black	x = 0	x = 0.25	x = 0.50	x = 0.75
Line	β=0.009(10)	β=0.15(1)	β=0.36(1)	β=0.78(30)
	$T_{N}=4.9(1)$	$T_{N}=6.20(1)$	$T_{N}=6.24(1)$	$T_{N}=6.7(5)$

Green	β=0.02(1)	β=0.155(8)	β=0.313(3)	For inset fit
Line	$T_{N}=4.97(5)$	$T_{N}=6.27(2)$	$T_{N}=6.42(2)$	β=0.33(15)
	Γ=0.58(2)	Γ=0.69(7)	Γ=0.35(7)	$T_{N}=5.63(5)$

	$T_{C}\sim 5\;K$	$T_{C}\sim 7.5\;K$	$T_{C}\sim 8\;K$	$T_{C}\sim 8.5\;K$
